# Photon diagnostics at the FLASH THz beamline^1^

**DOI:** 10.1107/S1600577519003412

**Published:** 2019-04-26

**Authors:** Rui Pan, Ekaterina Zapolnova, Torsten Golz, Aleksandar J. Krmpot, Mihailo D. Rabasovic, Jovana Petrovic, Vivek Asgekar, Bart Faatz, Franz Tavella, Andrea Perucchi, Sergey Kovalev, Bertram Green, Gianluca Geloni, Takanori Tanikawa, Mikhail Yurkov, Evgeny Schneidmiller, Michael Gensch, Nikola Stojanovic

**Affiliations:** a Deutsches Elektronen-Synchrotron (DESY), Notkestrasse 85, D-22607 Hamburg, Germany; b Institute of Physics Belgrade, Pregrevica 118, 11080 Belgrade, Serbia; c Vinca Institute of Nuclear Sciences, Belgrade, Serbia; d Center for Free-Electron Laser Science, Deutsches Elektronen-Synchrotron (DESY), Notkestrasse 85, D-22607 Hamburg, Germany; e Department of Physics, S. P. Pune University, Pune, India; f SLAC National Accelerator Laboratory, Menlo Park, California, USA; g Elettra – Sincrotrone Trieste SCpA, 34149 Basovizza, Trieste, Italy; h Helmholtz-Zentrum Dresden-Rossendorf (HZDR), Bautzner Landstraße 400, 01328 Dresden, Germany; i European XFEL, Holzkoppel 4, 22869 Schenefeld, Germany; j German Aerospace Center (DLR), Institute of Optical Sensor Systems, Rutherfordstraße 2, 12489 Berlin, Germany; k Institute of Optics and Atomic Physics, Technical University of Berlin, Strasse des 17 Juni 135, 10623 Berlin, Germany

**Keywords:** FLASH, intense THz, THz diagnostic, electro-optic, FTIR

## Abstract

The THz beamline at FLASH, DESY, provides both tunable (1–300 THz) narrow-bandwidth (∼10%) and broad-bandwidth intense (up to 150 uJ) THz pulses delivered in 1 MHz bursts and naturally synchronized with free-electron laser X-ray pulses. Combination of these pulses, along with the auxiliary NIR and VIS ultrashort lasers, supports a plethora of dynamic investigations in physics, material science and biology. The unique features of the FLASH THz pulses and the accelerator source, however, bring along a set of challenges in the diagnostics of their key parameters: pulse energy, spectral, temporal and spatial profiles. Here, these challenges are discussed and the pulse diagnostic tools developed at FLASH are presented. In particular, a radiometric power measurement is presented that enables the derivation of the average pulse energy within a pulse burst across the spectral range, jitter-corrected electro-optical sampling for the full spectro-temporal pulse characterization, spatial beam profiling along the beam transport line and at the sample, and a lamellar grating based Fourier transform infrared spectrometer for the on-line assessment of the average THz pulse spectra. Corresponding measurement results provide a comprehensive insight into the THz beamline capabilities.

## Abstract 2

A range of THz diagnostic tools developed for THz/XUV pump–probe experiments at FLASH1, DESY, are presented.

## 1. Introduction   

FLASH, the free-electron laser (FEL) in Hamburg at DESY, provides ultrafast XUV and soft X-ray radiation for users to perform pump–probe experiments. FLASH has two independent FEL undulator beamlines (Faatz *et al.*, 2016 ▸): FLASH1 and FLASH2. Each FEL branch ends with a dedicated experimental hall that has a number of beamlines.

**Figure 1:**
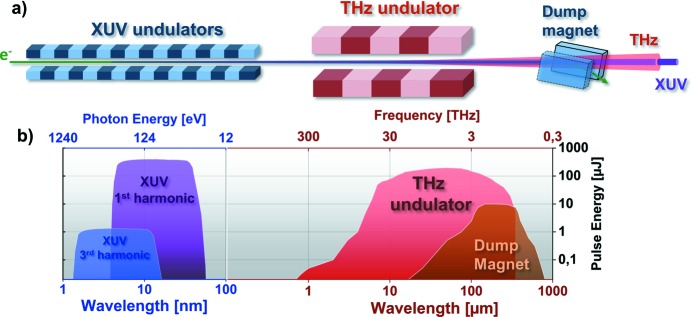
Scheme of the FLASH1 THz photon sources. (*a*) The THz undulator is located downstream of the XUV undulators, separated by free space. The electron beam dump magnet follows the THz undulator. (*b*) Representation of the pulse energies that can be obtained at FLASH1 from the XUV and THz sources over a wide spectral range.

FLASH1 has a unique feature, a dedicated THz undulator installed downstream of the XUV undulators. This feature allows the generation of intense THz pulses by the same electron bunch that generates XUV pulses (Stojanovic & Drescher, 2013 ▸), as shown in Fig. 1 ▸. As THz and XUV undulators are separated by empty drift space, XUV and THz pulses generated by the same electron bunch are naturally synchronized with no more than 5 fs timing jitter (Frühling *et al.*, 2009 ▸). Furthermore, THz pulses are carrier envelope phase (CEP) stable. Downstream of the THz undulator, the electron beam is deflected to ground by the so-called electron beam dump magnet (hereon referred to as the dump magnet). This stage separates the electron beam from the photon (THz and XUV) beams. The dump magnet on its own generates an intense THz transient, mainly by the edge and bending radiation process (Tavella *et al.*, 2011 ▸; Geloni *et al.*, 2009*a* ▸,*b* ▸). Only a fraction of the total bending radiation is collected in the THz beamline downstream (estimated to be 11.4%), as beamline design is optimized for the radiation in the forward direction, while bending radiation is created tangentially along the bend. THz and XUV beams are separated by a large flat mirror (210 mm × 140 mm) with a 10 mm aperture for the XUV beam (Gensch *et al.*, 2008 ▸). Transport of the THz beam into the experimental hall over ∼70 m requires multiple collimations and this is provided by all-reflective optics. By this unique photon generation scheme, the photon spectrum of FLASH1 is extended to the long-wavelength range. As shown in Fig. 1 ▸, FLASH1 covers the XUV range from 1.4 nm to 52 nm including harmonics (Tiedtke *et al.*, 2009 ▸), and the THz range from 1 µm to above 300 µm (300 THz to 1 THz). With an independent and synchronized near-infrared (NIR) laser (Redlin *et al.*, 2011 ▸) having a center wavelength of 800 nm, FLASH1 can provide XUV, THz and NIR laser beams for users at the same time to study photon–matter interactions.

**Figure 2:**
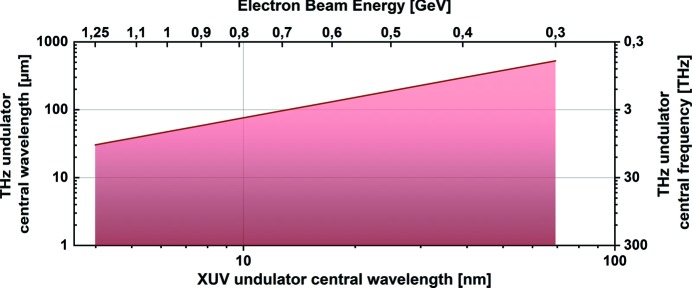
THz undulator spectral range. The shaded area represents the range where the fundamental frequency of the THz undulator radiation can be reached for FLASH1 as a function of the FEL XUV wavelength (lower horizontal axis) and the electron beam energy in the linac (upper horizontal axis).

Based on the scheme shown in Fig. 1 ▸, there are two types of intense THz sources. The first is a THz undulator (Grimm *et al.*, 2010 ▸) that generates tunable, linearly polarized (horizontally), narrow-bandwidth (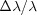 = 10%) radiation. The wavelength is tunable from 1 µm to above 300 µm. The longest wavelength that can be reached depends on the electron beam energy for a given THz undulator period and peak field (see Fig. 2 ▸). Pulse energies delivered to the experiment can reach up to 150 µJ, depending on the FLASH accelerator parameters (mainly the electron bunch charge and its compression).

The second source of THz radiation is the dump magnet that generates edge and bending radiation. The edge radiation is generated by the longitudinal acceleration of the electron beam at the interface between the free space and the dump magnet magnetic field (Tavella *et al.*, 2011 ▸; Geloni *et al.*, 2009*a* ▸,*b* ▸). This kind of radiation has a broad spectral bandwidth (quasi single-cycle temporal profile), is radially polarized and generated in the forward direction to the electron beam propagation. Electrons also generate the bending radiation along the bending arc of the dump magnet. The dump magnet vacuum chamber acceptance angle for the bending radiation is relatively small (2.4°) compared with 21° of the complete bend that the electrons experience. Thus only a fraction of the bending radiation radiated in the forward radiation is collected into the THz beamline. Also, the bending radiation has a broad bandwidth (quasi single-cycle temporal profile) and is linearly polarized. The bending radiation is collected by the THz beamline mostly from the bending plane and is thus polarized mainly in the vertical direction, orthogonal to the THz undulator pulse polarization. Combined edge and bending dump magnet radiation can reach a pulse energy of over 10 µJ. This radiation is generated parasitically and can be used independently from the undulator radiation.

**Figure 3:**
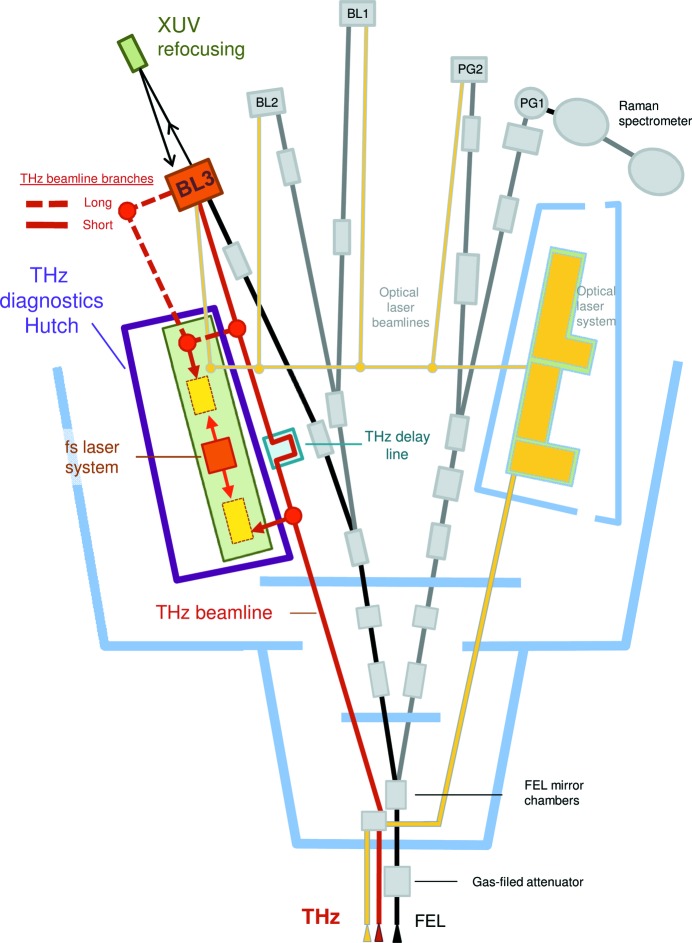
Scheme of the THz beamline in the FLASH1 experimental hall. THz beam is delivered to the end-station at the BL3 XUV beamline, via one of the two branches.

The THz beamline delivers the beam to the end-station at the end of the BL3 XUV beamline in the FLASH1 experimental hall (see Fig. 3 ▸). The THz beam can be delivered to the experiment via two branches, a short one with ultra-high-vacuum transport (10^−9^ mbar) and a long one via THz diagnostics hutch with high-vacuum transport (10^−7^ mbar). Due to the difference in optical path, the THz pulse arrives later than the XUV pulse to the end-station, 12 ns for the short branch and 21 ns for the long branch. We use two approaches to achieve temporal overlap of the XUV and THz pulses in the experiment: the first is delaying the XUV pulse by refocusing via multilayer mirrors; the second is to generate two electron bunches at the FLASH electron gun timed to achieve temporal overlap of the respective THz and XUV pulses in the experiment (Zapolnova *et al.*, 2018 ▸).

There are many unique applications for the CEP stable intrinsically synchronized THz pulses from the FLASH THz beamline. One important scientific area is in atomic and molecular physics. Here the THz field can act as a streak camera, allowing molecular reactions to be clocked and processes induced by the femtosecond-long XUV pulses from FLASH on a timescale of a few femtoseconds (Frühling *et al.*, 2009 ▸; Schütte *et al.*, 2012 ▸; Oelze *et al.*, 2017 ▸; Schmid *et al.*, 2019 ▸). An emerging new class of experiments at FLASH is the application of the strong THz fields for these tunable narrow-band pulses in selective excitation or selective THz control of matter [for a description of this field see, for example, Green *et al.* (2016 ▸), Buzzi *et al.* (2018 ▸), Kampfrath *et al.* (2013 ▸) and Kovalev *et al.* (2017 ▸)]. The first experiments performed at FLASH have focused on driving the magnetization dynamics in magnetic thin films by selective phonon excitation (Radu, 2019 ▸) and on the THz control of dynamic surface processes (Waltar *et al.*, 2018 ▸).

## 2. THz diagnostics   

In a typical THz-pump/XUV-probe experiment at FLASH, determination of the properties of the driving THz pulse is of key importance. Based on the needs of the user experiments in past years, we have developed diagnostics tools to fully characterize the THz beam at the experiment. Presently, all the tools are developed in the THz diagnostics hutch at FLASH1 experimental hall and will be transferred to the end-station at BL3 beamline (see Fig. 3 ▸). In this paper we present tools for the full spectral, temporal and spatial characterization of the THz pulses and the pulse-energy measurement. Thereby we discuss some of the major challenges for diagnostics, *i.e.* the extremely broad THz spectral range of FLASH sources, 1 MHz repetition rate in 10 Hz bursts and jitter to externally synchronized lasers (Azima *et al.*, 2009 ▸; Tavella *et al.*, 2011 ▸), that can be used for THz waveform characterization.

### 2.1. THz power measurement   

We measure the THz pulse energies using a radiometer (RM3700, head RjP-735/RF, by Laser Probe). We have cross-referenced this detector to a PTB (The National Metrology Institute of Germany) traceable 3A-P-THz, by Ophir Optronics Solutions (Green *et al.*, 2016 ▸). The radiometer detector has a cavity pyroelectric probe and it has a time constant of 1 ms. Its temporal response prevents us from resolving individual pulses of the FLASH micro-pulses within a 1 MHz burst. However, the detector time constant is well matched to the maximal duration of the 1 MHz burst (with duration of 0.8 ms, containing up to 800 pulses) defined by the FLASH accelerator. Therefore, the integration is performed over all micro-bunches in a burst, which allows for determination of the average THz pulse energy with good accuracy.

**Figure 4:**
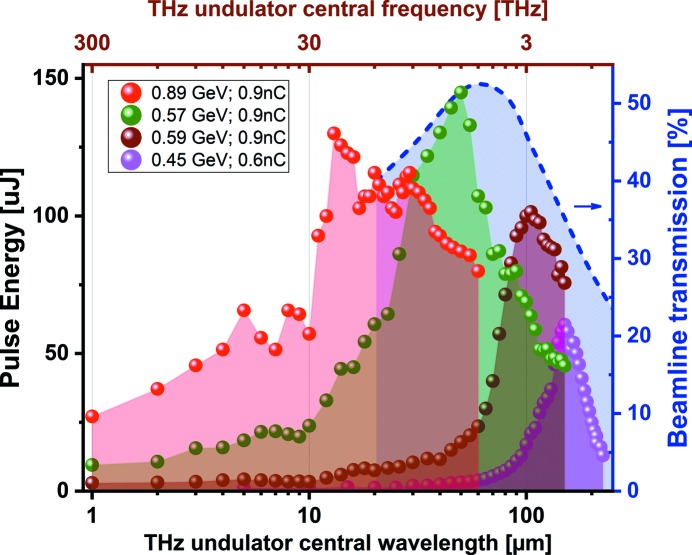
THz pulse energies measured at the beamline end-station for different conditions of the FLASH accelerator. The blue dashed line shows the beamline transmission, calculated using the *SRW* software package (Chubar & Elleaume, 1998 ▸).

Fig. 4 ▸ depicts the THz pulse energy as a function of the central wavelength of the THz undulator. The total THz beamline transmission (from the source until the end of beamline) as a function of wavelength is depicted as well. We calculate the beamline transmission by modeling the THz source and the optical transport with the *Synchrotron Radiation Workshop* (*SRW*) software package (Chubar & Elleaume, 1998 ▸). We also account for the Fresnel losses in the diamond window that separates the beamline from the accelerator vacuum (Gensch *et al.*, 2008 ▸). We present examples of four measurements taken for different electron beam settings of the FLASH accelerator. Note that the abrupt end of the THz undulator tuning range at long wavelengths relates to the maximal wavelength that can be reached at a particular electron beam energy and is determined by the maximum field (1.2 T) inside the undulator (see also Fig. 2 ▸).

### 2.2. THz temporal profile measurements   

The THz time domain spectroscopy (TDS) via electro-optic sampling (EOS) method is a well established technique for the full characterization of the THz pulse temporal structure (Wu & Zhang, 1995 ▸; Schmuttenmaer, 2004 ▸), in a broad spectral range. The electric field of the THz pulse changes the birefringence of the EOS crystal. This transient change is sampled by an ultrashort laser pulse. By scanning the laser pulse in time, the complete THz pulse shape can be reconstructed.

At an accelerator-based light source, such as FLASH, the laser system is synchronized to the master clock of the FLASH accelerator (Schulz *et al.*, 2015 ▸). One of the main limiting factors for the use of an externally synchronized laser for EOS detection is the temporal jitter between the FEL and the laser pulses. Jitter limits the temporal resolution and subsequently the spectral bandwidth of EOS detection. We have measured the jitter of the probe laser (pulse duration 20 fs FWHM) in the THz hutch to the FLASH THz pulses to be around 100 fs RMS (∼200 fs peak-to-peak). To solve this, we chose to detect the THz pulses’ arrival time on a single-shot basis using spectral decoding electro-optic detection (EOSD) (Jiang & Zhang, 1998 ▸). This technique enables single-shot THz detection by imprinting the THz pulse electric field onto a stretched probe laser pulse, thus defining the arrival time of one with respect to the other. Spectral decoding is photon efficient (for a single-shot method) and enables the arrival time detection even with femtosecond oscillator pulses, which allows for a high-repetition-rate arrival time detection scheme that can be matched to the FLASH pulse pattern. Full EO detection of the THz pulses at FLASH then comprises two main components: arrival time monitoring by spectral decoding in combination with scanning electro-optic sampling (EOS). EOS data are sorted for their arrival time and the THz pulse shape is retrieved. The complete detection setup is installed in a high-vacuum chamber (10^−7^ mbar) to avoid measurement distortions by absorption in ambient air. We achieved a temporal resolution of the arrival time sorting of 9.7 fs RMS. Most importantly, the EOS sampling has a bandwidth of 37–3000 µm (0.1–8 THz) limited by the gallium phosphide (GaP) EOS crystal (100 µm thick). The setup has been developed in collaboration with the TELBE team at HZDR and details can be found in the literature (Kovalev *et al.*, 2017 ▸; Golz, 2018 ▸).

**Figure 5:**
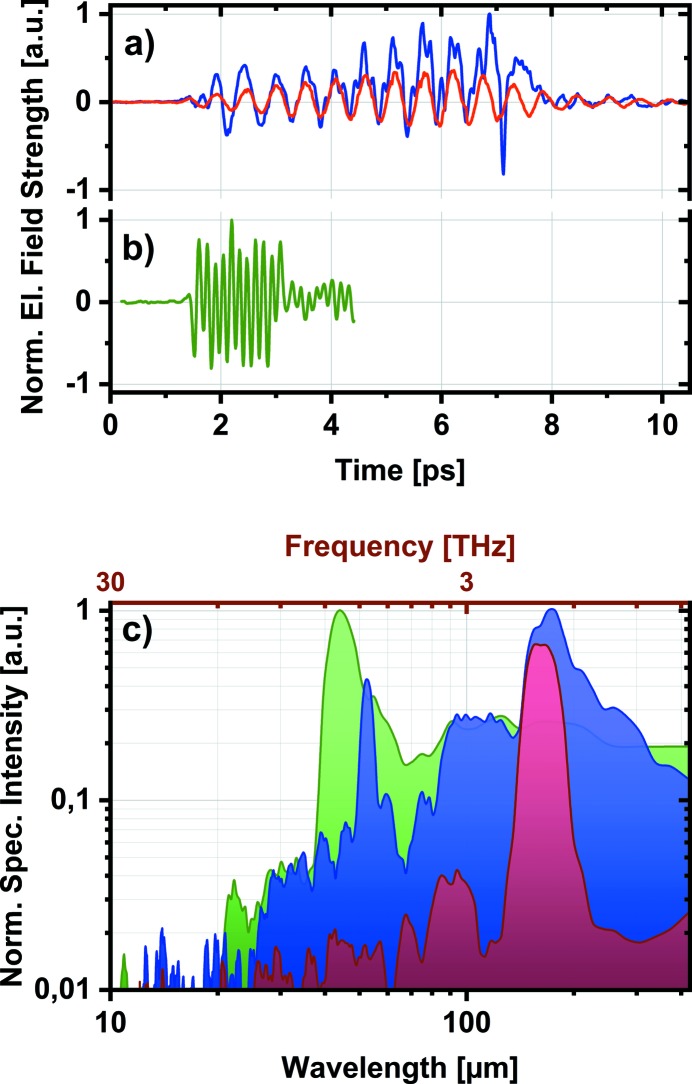
THz pulse waveforms measured by arrival-time-sorted time domain spectroscopy (TDS). (*a*) The THz pulse produced by the undulator set at the nominal wavelength of 155 µm (1.93 THz). The unfiltered temporal profile is shown by the blue line and the filtered by the red line, with 155 µm (1.93 THz) bandpass filter (15% bandwidth). (*b*) The unfiltered THz pulse at 43 µm (7 THz) (green line). (*c*) The corresponding THz pulse spectra.

Two typical examples of measured THz waveform profiles are shown in Figs. 5(*a*) and 5(*b*) ▸. The THz and the probing laser beam are overlapped and focused on the GaP EOS crystal, with beam sizes of 350 µm FWHM and 70 µm FWHM, respectively. The THz undulator was set to nominal wavelengths of 155 µm and 42 µm (corresponding to frequencies of 1.93 THz and 7.1 TH, respectively). For the long-wavelength example, unfiltered and spectrally filtered pulses are presented. The wire-grid THz bandpass filter used has been centered at 155 µm wavelength (1.93 THz) with 15% spectral bandwidth. For the unfiltered pulse, it is interesting to observe that the electric field and high-harmonic content increase along the pulse. This indicates the change of the electron bunch form factor (Nodvick & Saxon, 1954 ▸) inside the undulator. In the respective unfiltered THz pulse spectrum [see Fig. 5(*c*) ▸] we clearly observe the first harmonic peaking at 169 µm (1.77 THz) and the third harmonic at 52 µm (5.68 THz), and the baseline includes the broadband spectrum from the dump magnet radiation. As expected, the measurement with the THz bandpass filter shows a strong peak around 160 µm (1.87 THz), with a small (few percent) leakage between 75 and 100 µm (3 and 4 THz). Similarly, in the spectrum of the short-wavelength pulse (tuned to 42 µm) we observe the first harmonic peaking at 43 µm (7 THz).

It is worth noting that the EOSD scheme can be used as a THz arrival-time detection tool in other experiments. A recent application evaluated the timing jitter between two electron bunches with 21 ns delay timed for temporal overlap of THz and XUV pulses at the experimental end-station at BL3 (Zapolnova *et al.*, 2018 ▸).

### 2.3. THz beam profile   

Knowledge of the transverse THz beam profile is important as it allows precise determination of the fluence (and the peak field) on the sample, as well as optimal optical design to maximize the beam transmission in the experiment. We model the THz source and the radiation transport using the *SRW* software package (Chubar & Elleaume, 1998 ▸) and observe the strong interference effects between undulator and dump magnet radiation (edge and bending radiation) at FLASH (Asgekar *et al.*, 2014 ▸).

**Figure 6:**
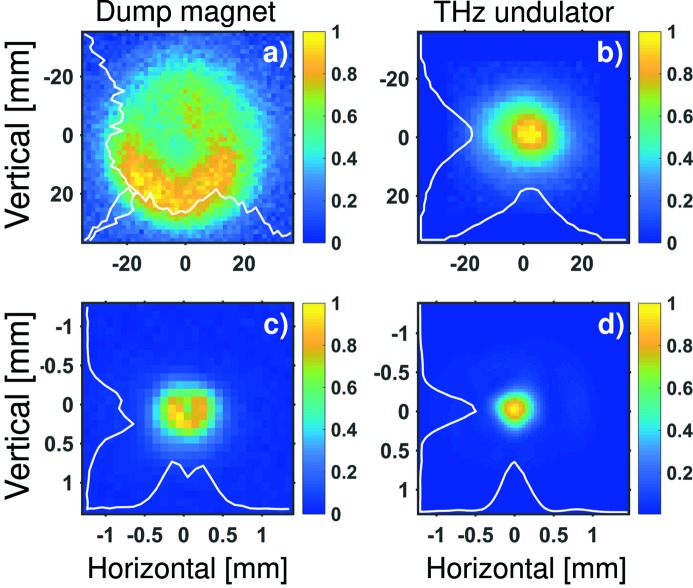
(Top) THz transverse beam profiles at FLASH. (*a*) Dump magnet (the THz undulator is off), and (*b*) THz undulator tuned to 88 µm (3.4 THz), unfocused beam profiles measured in the THz hutch; beam position imaged 10 m from the virtual source. Note that the undulator profile is padded with zeros to fill in the same image size. (Bottom) The same beams focused. (*c*) Dump magnet beam (600 µm FWHM), and (*d*) THz undulator beam (350 µm FWHM).

An example of the measured THz beam profile from the dump magnet, with the THz undulator switched off, is shown in Fig. 6(*a*) ▸. An example of the THz undulator beam tuned to 88 µm (3.4 THz) is shown in Fig. 6(*b*) ▸. The beams have been imaged in the THz diagnostics hutch so that they image the plane approximately 10 m downstream of the radiation source. We perform 2D raster scanning of the beams. As a detector we employ an amplified pyroelectric detector (LME-501 from InfraTec). Both measurements have been performed with 30 µm long-pass THz spectral filter. The profiles have been reproduced by *SRW* calculation, whereby the integration of the output power was performed over the 30–300 µm (1–10 THz) spectral range to account for the filtering, and the form factor of a 50 fs RMS long electron bunch was used.

For the dump magnet, both the measurement and calculation render a half-moon-like structure that can be explained by interference of the edge radiation with the bending radiation from the dump magnet. For the THz undulator beam we observe slight asymmetry in the horizontal plane which can be explained by interference of the undulator and the dump magnet radiation (Asgekar *et al.*, 2014 ▸).

We focus both beams with an off-axis parabolic mirror of focal length 150 mm and measure the beam profile with a Pyrocam III camera from Ophir Photonics for the dump magnet and with a microbolometer camera for the undulator beam. The results are shown in Figs. 6(*c*) and 6(*d*) ▸, respectively. We observe a beam size of 600 µm FWHM for the dump magnet and 350 µm FWHM for the undulator beam.

**Figure 7:**
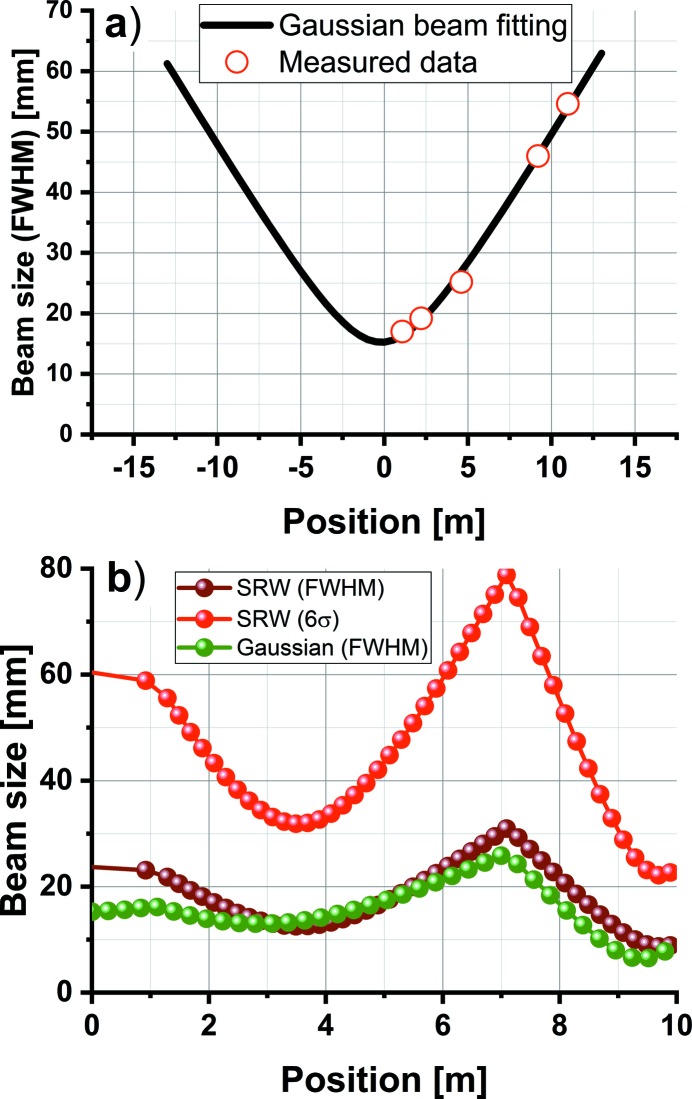
THz undulator beam size at 160 µm (1.87 THz) and its propagation. (*a*) Beam size measured at five different locations along the beam path (red circles) fitted by a Gaussian beam propagation. (*b*) THz beam propagation over the 10 m path, with the focusing mirrors inserted at the 1 m and 7 m position marks. The THz beam was approximated by fitted Gaussian beam (FWHM) (green curve) and calculated from the source by *SRW* (FWHM) (brown curve) and 6σ (red curve).

Moreover, we follow the THz undulator beam profile evolution, by wavefront propagation in *SRW*. The undulator radiation wavelength is set to 160 µm (1.87 THz). For an experimental confirmation, we measure the beam profile in the THz diagnostics hutch at five different positions along the beam path [see Fig. 7(*a*) ▸], using the knife-edge technique. Zero position denotes the THz beamline window at which the beam is extracted into the THz diagnostics station. We assume a Gaussian beam profile and obtain the beam parameters by fitting. The plot in Fig. 7(*b*) ▸ compares the beam size evolution (FWHM) calculated by *SRW* (brown curve) with the approximated Gaussian beam propagation (ABCD matrix formalism), fitted from the measured data (green curve), for the last 10 m of the THz beamline transport. Two toroidal mirrors with focal lengths of 3.8 m and 1.8 m are inserted at the 1 m and 7 m position marks, respectively. In this particular example, the goal was to couple the THz beam into an experimental chamber through a 25 mm aperture, located at the 10 m position mark, with highest possible transmission. The red curve depicts the THz beam size at the equivalent of 6σ (or 99.7% beam energy), and at the 10 m position mark we achieve the desired sub-25 mm beam size. We observe a reasonable agreement between these two numerical models, with the big advantage of the Gaussian beam propagation providing very fast and efficient evaluation of the beam sizes in the optical system.

### 2.4. THz spectrum measurement   

For a quick THz spectral characterization, when temporal pulse structure is not necessary, we have developed the variation of the Fourier transform infrared (FTIR) spectrometer based on a reflective lamellar grating. Unlike the most commonly used Michelson interferometer (based on amplitude division), the reflective lamellar grating interferometer (Richards, 1964 ▸; Bell, 1972 ▸) divides the wavefront spatially. Because of this, the reflective lamellar grating design has a key advantage: a high (close to 100%) and smooth efficiency response (*e.g.* typical Fabry–Perot interferences that plague Michelson interferometers are absent). A comparison of the efficiency of the lamellar grating and the Michelson interferometer can be found in Fig. 3 of Richards (1964 ▸). As a side note, owing to a large spectral bandwidth, the lamellar gratings have found application in extreme ultraviolet (XUV) spectroscopy and metrology (Gebert *et al.*, 2014 ▸; Usenko *et al.*, 2017 ▸).

**Figure 8:**
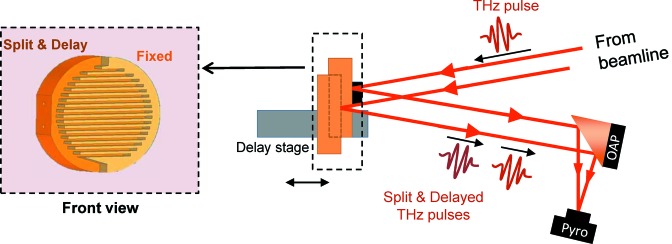
Scheme of the lamellar grating interferometer. OAP: off-axis parabolic mirror.

As shown in Fig. 8 ▸, the lamellar grating spectrometer consists of two interleaved gratings, manufactured from a 100 mm-diameter gold-coated copper mirror. One is fixed and the other is mounted on the motorized stage responsible for introducing the optical delay between the split beams. For detection a pyroelectric detector (LME-501 from InfraTec) with a 2 mm × 2 mm chip size was used.

The THz beam is collimated in a way that uniformly illuminates the gratings. The period of the grating (*h* = 2 mm) is chosen to match the spectral range of the THz sources at FLASH. The long-wavelength limit (Bell, 1972 ▸) for lamellar gratings is λ_max_ < *h*/2, which is 1 mm (corresponding to 0.3 THz) in our case. For wavelengths longer than λ_max_, a cavity effect starts decreasing the modulation depth of the THz waves, which are polarized parallel to the fringes. The high-frequency limit for this geometry is 30 µm (corresponding to 10 THz), which is determined by diffraction theory and depends on the geometry of the device (Strong & Vanasse, 1960 ▸; Naftaly *et al.*, 2008 ▸; Ferhanoglu *et al.*, 2009 ▸): λ_min_ = *hs*/*f*, where *f* = 130 mm is the focal length of the parabolic mirror and *s* = 2 mm is the width of the exit slit of the detector (defined by the detector effective aperture).

**Figure 9:**
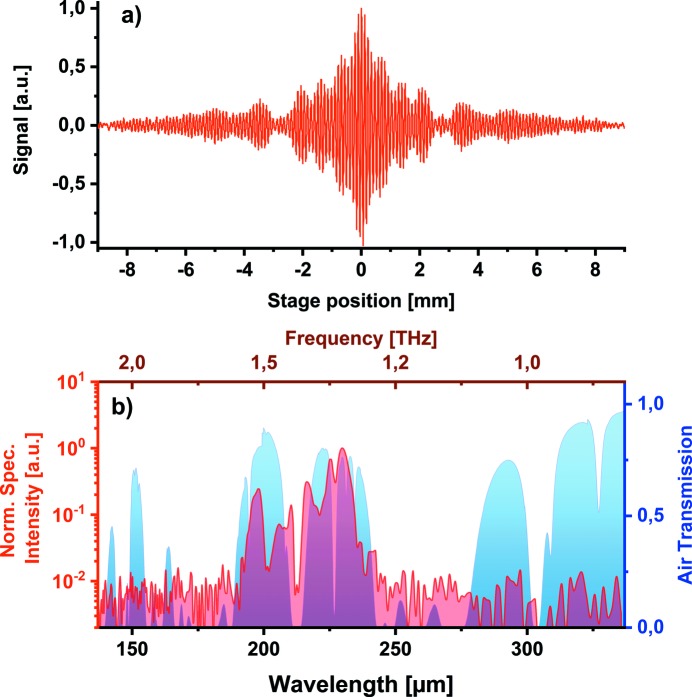
FTIR measurement for THz edge radiation with 215 µm (1.4 THz) THz bandpass filter. (*a*) Double-sided interferogram. (*b*) Respective spectrum (red curve) and air transmission with water vapor (in blue) show the strong absorption lines in this spectral range.

An example of the measured interferogram and the calculated spectrum is shown in Fig. 9 ▸. The THz pulse was generated by the edge radiation and filtered by a 215 µm (1.4 THz) bandpass filter. The fluctuations of the shot-to-shot THz pulse energy at FLASH can be as high as 20% RMS, depending on the FLASH accelerator settings. We split a small portion of the beam for a reference measurement that is then used to normalize the measured interferogram on a single-shot basis. The normalized interferogram in Fig. 9(*a*) ▸ was obtained during 20 min of scanning at 10 Hz repetition rate (12000 shots). Fig. 9(*b*) ▸ shows the normalized spectrum, obtained by taking a Fourier transform of the measured interferogram. We observe the spectral content as expected from the filter response with a signal-to-noise ratio exceeding 100.

Measurement has been performed in ambient air and we observe a strong modulation from the water vapor absorption lines. We are currently upgrading it to an all-in-vacuum spectrometer.

## 3. Conclusion   

We have presented a range of THz diagnostic tools developed for THz/XUV pump–probe experiments at FLASH1, DESY. The THz pulse energy is an important parameter for optimization of the FLASH accelerator, and it reaches values from tens of µJ up to 150 µJ. The upgrade of a currently used radiometer to an online monitor is in progress. The THz temporal profile can be measured with 10 fs timing resolution and covers the spectral range from 37 to 3000 µm (0.1 to 8 THz). The ongoing development of this technique will explore the use of different EOS crystals to extend the measured bandwidth to shorter wavelengths, GaSe (Kübler *et al.*, 2005 ▸) and SiC (Naftaly *et al.*, 2016 ▸) being good candidates that should allow THz detection in the 10–40 THz and 0.1–15 THz spectral windows, respectively. To fully exploit the high THz pulse energies at FLASH, we are upgrading the single-shot EOSD THz detection technique to one via tilted laser pulse front (Teo *et al.*, 2015 ▸). This technique is free from the spectral distortions that plague EOSD (Jamison *et al.*, 2008 ▸) and allows for the full bandwidth of the pulse to be retrieved (limited only by the EOS crystal). The measurements of the transversal THz beam profile are used for the design of the beam transfer line and calibration of the peak field and intensity in the experiment. A broadband FTIR spectrometer, covering the spectral range 30–1000 µm (0.3–10 THz), based on a reflective lamellar grating is developed for spectral measurements. It will be permanently installed inside the THz beamline vacuum environment for distortion-free THz spectrum measurements, enabling quick and robust spectral studies [*e.g.* suitable for THz shaping by emerging THz meta-materials (Yen *et al.*, 2004 ▸; Monticone & Alù, 2017 ▸; Stojanović *et al.*, 2018 ▸; Polley *et al.*, 2018 ▸)]. 

## References

[bb1] Asgekar, V., Geloni, G., Kocharyan, V., Stojanovic, N., Michel, P. & Gensch, M. (2014). *Infrared Phys. Technol.* **64**, 26–32.

[bb2] Azima, A., Düsterer, S., Radcliffe, P., Redlin, H., Stojanovic, N., Li, W., Schlarb, H., Feldhaus, J., Cubaynes, D., Meyer, M., Dardis, J., Hayden, P., Hough, P., Richardson, V., Kennedy, E. T. & Costello, J. T. (2009). *Appl. Phys. Lett.* **94**, 144102.

[bb3] Bell, R. J. (1972). *Introductory Fourier Transform Infrared Spectroscopy.* New York: Academic Press.

[bb4] Buzzi, M., Först, M., Mankowsky, R. & Cavalleri, A. (2018). *Nat. Rev. Mater.* **3**, 299–311.

[bb5] Chubar, O. & Elleaume, P. (1998). *Proceedings of the Sixth European Particle Accelerator Conference (EPAC’98)*, 22–26 June 1998, Stockholm, Sweden, pp. 1177–1179.

[bb6] Faatz, B., Plönjes, E., Ackermann, S., Agababyan, A., Asgekar, V., Ayvazyan, V., Baark, S., Baboi, N., Balandin, V., Bargen, N., Bican, Y., Bilani, O., Bödewadt, J., Böhnert, M., Böspflug, R., Bonfigt, S., Bolz, H., Borges, F., Borkenhagen, O., Brachmanski, M., Braune, M., Brinkmann, A., Brovko, O., Bruns, T., Castro, P., Chen, J., Czwalinna, M. K., Damker, H., Decking, W., Degenhardt, M., Delfs, A., Delfs, T., Deng, H., Dressel, M., Duhme, H., Düsterer, S., Eckoldt, H., Eislage, A., Felber, M., Feldhaus, J., Gessler, P., Gibau, M., Golubeva, N., Golz, T., Gonschior, J., Grebentsov, A., Grecki, M., Grün, C., Grunewald, S., Hacker, K., Hänisch, L., Hage, A., Hans, T., Hass, E., Hauberg, A., Hensler, O., Hesse, M., Heuck, K., Hidvegi, A., Holz, M., Honkavaara, K., Höppner, H., Ignatenko, A., Jäger, J., Jastrow, U., Kammering, R., Karstensen, S., Kaukher, A., Kay, H., Keil, B., Klose, K., Kocharyan, V., Köpke, M., Körfer, M., Kook, W., Krause, B., Krebs, O., Kreis, S., Krivan, F., Kuhlmann, J., Kuhlmann, M., Kube, G., Laarmann, T., Lechner, C., Lederer, S., Leuschner, A., Liebertz, D., Liebing, J., Liedtke, A., Lilje, L., Limberg, T., Lipka, D., Liu, B., Lorbeer, B., Ludwig, K., Mahn, H., Marinkovic, G., Martens, C., Marutzky, F., Maslocv, M., Meissner, D., Mildner, N., Miltchev, V., Molnar, S., Mross, D., Müller, F., Neumann, R., Neumann, P., Nölle, D., Obier, F., Pelzer, M., Peters, H., Petersen, K., Petrosyan, A., Petrosyan, G., Petrosyan, L., Petrosyan, V., Petrov, A., Pfeiffer, S., Piotrowski, A., Pisarov, Z., Plath, T., Pototzki, P., Prandolini, M. J., Prenting, J., Priebe, G., Racky, B., Ramm, T., Rehlich, K., Riedel, R., Roggli, M., Röhling, M., Rönsch-Schulenburg, J., Rossbach, J., Rybnikov, V., Schäfer, J., Schaffran, J., Schlarb, H., Schlesselmann, G., Schlösser, M., Schmid, P., Schmidt, C., Schmidt-Föhre, F., Schmitz, M., Schneidmiller, E., Schöps, A., Scholz, M., Schreiber, S., Schütt, K., Schütz, U., Schulte-Schrepping, H., Schulz, M., Shabunov, A., Smirnov, P., Sombrowski, E., Sorokin, A., Sparr, B., Spengler, J., Staack, M., Stadler, M., Stechmann, C., Steffen, B., Stojanovic, N., Sychev, V., Syresin, E., Tanikawa, T., Tavella, F., Tesch, N., Tiedtke, K., Tischer, M., Treusch, R., Tripathi, S., Vagin, P., Vetrov, P., Vilcins, S., Vogt, M., Wagner, A. Z., Wamsat, T., Weddig, H., Weichert, G., Weigelt, H., Wentowski, N., Wiebers, C., Wilksen, T., Willner, A., Wittenburg, K., Wohlenberg, T., Wortmann, J., Wurth, W., Yurkov, M., Zagorodnov, I. & Zemella, J. (2016). *New J. Phys.* **18**, 062002.

[bb7] Ferhanoglu, O., Seren, H. R., Lüttjohann, S. & Urey, H. (2009). *Opt. Express*, **17**, 21289–21301. 10.1364/OE.17.021289 19997368

[bb8] Frühling, U., Wieland, M., Gensch, M., Gebert, T., Schütte, B., Krikunova, M., Kalms, R., Budzyn, F., Grimm, O., Rossbach, L., Plönjes, E. & Drescher, M. (2009). *Nat. Photon.* **3**, 523.

[bb9] Gebert, T., Rompotis, D., Wieland, M., Karimi, F., Azima, A. & Drescher, M. (2014). *New J. Phys.* **16**, 073047.

[bb10] Geloni, G., Kocharyan, V., Saldin, E., Schneidmiller, E. & Yurkov, M. (2009*a*). *Nucl. Instrum. Methods Phys. Res. A*, **605**, 409–429.

[bb11] Geloni, G., Kocharyan, V., Saldin, E., Schneidmiller, E. & Yurkov, M. (2009*b*). *Nucl. Instrum. Methods Phys. Res. A*, **607**, 470–487.

[bb12] Gensch, M., Bittner, L., Chesnov, A., Delsim-Hashemi, H., Drescher, M., Faatz, B., Feldhaus, J., Fruehling, U., Geloni, G., Gerth, C., Grimm, O., Hahn, U., Hesse, M., Kapitzki, S., Kocharyan, V., Kozlov, O., Matyushevsky, E., Morozov, N., Petrov, D., Ploenjes, E., Roehling, M., Rossbach, J., Saldin, E. L., Schmidt, B., Schmueser, P., Schneidmiller, E. A., Syresin, E., Willner, A. & Yurkov, M. V. (2008). *Infrared Phys. Technol.* **51**, 423–425.

[bb13] Golz, T. (2018). PhD thesis, Hamburg University, Germany.

[bb14] Green, B., Kovalev, S., Asgekar, V., Geloni, G., Lehnert, U., Golz, T., Kuntzsch, M., Bauer, C., Hauser, J., Voigtlaender, J., Wustmann, B., Koesterke, I., Schwarz, M., Freitag, M., Arnold, A., Teichert, J., Justus, M., Seidel, W., Ilgner, C., Awari, N., Nicoletti, D., Kaiser, S., Laplace, Y., Rajasekaran, S., Zhang, L., Winnerl, S., Schneider, H., Schay, G., Lorincz, I., Rauscher, A. A., Radu, I., Mährlein, S., Kim, T. H., Lee, J. S., Kampfrath, T., Wall, S., Heberle, J., Malnasi-Csizmadia, A., Steiger, A., Müller, A. S., Helm, M., Schramm, U., Cowan, T., Michel, P., Cavalleri, A., Fisher, A. S., Stojanovic, N. & Gensch, M. (2016). *Sci. Rep.* **6**, 22256. 10.1038/srep22256 PMC4770290 26924651

[bb15] Grimm, O., Morozov, N., Chesnov, A., Holler, Y., Matushevsky, E., Petrov, D., Rossbach, J., Syresin, E. & Yurkov, M. (2010). *Nucl. Instrum. Methods Phys. Res. A*, **615**, 105–113.

[bb16] Jamison, S., Gillespie, W. & Phillips, P. (2008). *Signal*, **1**, 2.

[bb17] Jiang, Z. & Zhang, X.-C. (1998). *Appl. Phys. Lett.* **72**, 1945–1947.

[bb18] Kampfrath, T., Tanaka, K. & Nelson, K. A. (2013). *Nat. Photon.* **7**, 680.

[bb19] Kovalev, S., Green, B., Golz, T., Maehrlein, S., Stojanovic, N., Fisher, A. S., Kampfrath, T. & Gensch, M. (2017). *Struct. Dyn.* **4**, 024301. 10.1063/1.4978042 PMC5346102 28382317

[bb20] Kübler, C., Huber, R. & Leitenstorfer, A. (2005). *Semicond. Sci. Technol.* **20**, S128–S133.

[bb21] Monticone, F. & Alù, A. (2017). *Rep. Prog. Phys.* **80**, 036401. 10.1088/1361-6633/aa518f 28166060

[bb22] Naftaly, M., Dean, P., Miles, R. E., Fletcher, J. R. & Malcoci, A. (2008). *Quantum Electron.* **14**, 443–448.

[bb23] Naftaly, M., Molloy, J., Magnusson, B., Andreev, Y. & Lanskii, G. (2016). *Opt. Express*, **24**, 2590–2595. 10.1364/OE.24.002590 26906831

[bb24] Nodvick, J. S. & Saxon, D. S. (1954). *Phys. Rev.* **96**, 180–184.

[bb25] Oelze, T., Schütte, B., Müller, M., Müller, J. P., Wieland, M., Frühling, U., Drescher, M., Al-Shemmary, A., Golz, T., Stojanovic, N. & Krikunova, M. (2017). *Sci. Rep.* **7**, 40736. 10.1038/srep40736 PMC5241628 28098175

[bb44] Polley, D., Hagström, N. Z., Schmising, C. K., Eisebitt, S. & Bonetti, S. (2018). *J. Phys. B At. Mol. Opt. Phys.* **51**, 224001.

[bb26] Radu, I. (2019). In preparation.

[bb27] Redlin, H., Al-Shemmary, A., Azima, A., Stojanovic, N., Tavella, F., Will, I. & Düsterer, S. (2011). *Nucl. Instrum. Methods Phys. Res. A*, **635**, S88–S93.

[bb28] Richards, P. L. (1964). *J. Opt. Soc. Am.* **54**, 1474–1484.

[bb29] Schmid, G., Schnorr, K., Augustin, S., Meister, S., Lindenblatt, H., Trost, F., Liu, Y., Stojanovic, N., Al-Shemmary, A., Golz, T., Treusch, R., Gensch, M., Kübel, M., Foucar, L., Rudenko, A., Ullrich, J., Schröter, C. D., Pfeifer, T. & Moshammer, R. (2019). *Phys. Rev. Lett.* **122**, 073001. 10.1103/PhysRevLett.122.073001 30848607

[bb30] Schmuttenmaer, C. A. (2004). *Chem. Rev.* **104**, 1759–1779. 10.1021/cr020685g 15080711

[bb31] Schulz, S., Grguraš, I., Behrens, C., Bromberger, H., Costello, J., Czwalinna, M., Felber, M., Hoffmann, M., Ilchen, M., Liu, H., Mazza, T., Meyer, M., Pfeiffer, S., Prędki, P., Schefer, S., Schmidt, C., Wegner, U., Schlarb, H. & Cavalieri, A. L. (2015). *Nat. Commun.* **6**, 5938. 10.1038/ncomms6938 PMC4309427 25600823

[bb32] Schütte, B., Bauch, S., Frühling, U., Wieland, M., Gensch, M., Plönjes, E., Gaumnitz, T., Azima, A., Bonitz, M. & Drescher, M. (2012). *Phys. Rev. Lett.* **108**, 253003. 10.1103/PhysRevLett.108.253003 23004594

[bb43] Stojanović, D. B., Beličev, P. P., Gligorić, G. & Hadžievski, L. (2018). *J. Phys. D Appl. Phys.* **51**, 045106.

[bb33] Stojanovic, N. & Drescher, M. (2013). *J. Phys. B At. Mol. Opt. Phys.* **46**, 192001.

[bb34] Strong, J. & Vanasse, G. A. (1960). *J. Opt. Soc. Am.* **50**, 113–118.

[bb35] Tavella, F., Stojanovic, N., Geloni, G. & Gensch, M. (2011). *Nat. Photon.*, **5**, 162.

[bb36] Teo, S. M., Ofori-Okai, B. K., Werley, C. A. & Nelson, K. A. (2015). *Rev. Sci. Instrum.* **86**, 051301. 10.1063/1.4921389 26026507

[bb37] Tiedtke, K., Azima, A., von Bargen, N., Bittner, L., Bonfigt, S., Düsterer, S., Faatz, B., Frühling, U., Gensch, M., Gerth, C., Guerassimova, N., Hahn, U., Hans, T., Hesse, M., Honkavaar, K., Jastrow, U., Juranic, P., Kapitzki, S., Keitel, B., Kracht, T., Kuhlmann, M., Li, W. B., Martins, M., Núñez, T., Plönjes, E., Redlin, H., Saldin, E. L., Schneidmiller, E. A., Schneider, J. R., Schreiber, S., Stojanovic, N., Tavella, F., Toleikis, S., Treusch, R., Weigelt, H., Wellhöfer, M., Wabnitz, H., Yurkov, M. V. & Feldhaus, J. (2009). *New J. Phys.* **11**, 023029.

[bb38] Usenko, S., Przystawik, A., Jakob, M. A., Lazzarino, L. L., Brenner, G., Toleikis, S., Haunhorst, C., Kip, D. & Laarmann, T. (2017). *Nat. Commun.* **8**, 15626. 10.1038/ncomms15626 PMC5459985 28555640

[bb39] Waltar, K., Haase, J., Lucchini, M., van Bokhoven, J. A., Hengsberger, M., Osterwalder, J. & Castiglioni, L. (2018). *Opt. Express*, **26**, 8364–8374. 10.1364/OE.26.008364 29715804

[bb40] Wu, Q. & Zhang, X. (1995). *Appl. Phys. Lett.* **67**, 3523–3525.

[bb41] Yen, T.-J., Padilla, W., Fang, N., Vier, D., Smith, D., Pendry, J., Basov, D. & Zhang, X. (2004). *Science*, **303**, 1494–1496. 10.1126/science.1094025 15001772

[bb42] Zapolnova, E., Golz, T., Pan, R., Klose, K., Schreiber, S. & Stojanovic, N. (2018). *J. Synchrotron Rad.* **25**, 39–43. 10.1107/S1600577517015442 PMC5741119 29271749

